# Causal relationships involving brain imaging-derived phenotypes based on UKB imaging cohort: a review of Mendelian randomization studies

**DOI:** 10.3389/fnins.2024.1436223

**Published:** 2024-07-10

**Authors:** Mengdong Wang, Zirui Wang, Yaoyi Wang, Quan Zhou, Junping Wang

**Affiliations:** ^1^Department of Radiology, The Third Affiliated Hospital of Southern Medical University, Guangzhou, China; ^2^Department of Radiology and Tianjin Key Laboratory of Functional Imaging, Tianjin Medical University General Hospital, Tianjin, China

**Keywords:** Mendelian randomization, neuroimaging, IDPs, UKB, MR

## Abstract

The UK Biobank (UKB) has the largest adult brain imaging dataset, which encompasses over 40,000 participants. A significant number of Mendelian randomization (MR) studies based on UKB neuroimaging data have been published to validate potential causal relationships identified in observational studies. Relevant articles published before December 2023 were identified following the PRISMA protocol. Included studies (*n* = 34) revealed that there were causal relationships between various lifestyles, diseases, biomarkers, and brain image-derived phenotypes (BIDPs). In terms of lifestyle habits and environmental factors, there were causal relationships between alcohol consumption, tea intake, coffee consumption, smoking, educational attainment, and certain BIDPs. Additionally, some BIDPs could serve as mediators between leisure/physical inactivity and major depressive disorder. Regarding diseases, BIDPs have been found to have causal relationships not only with Alzheimer’s disease, stroke, psychiatric disorders, and migraine, but also with cardiovascular diseases, diabetes, poor oral health, osteoporosis, and ankle sprain. In addition, there were causal relationships between certain biological markers and BIDPs, such as blood pressure, LDL-C, IL-6, telomere length, and more.

## Introduction

The importance of establishing causality in scientific data analysis cannot be overstated, as it involves determining the true cause-effect relationship between variables. Randomized controlled trials (RCTs) are the gold standard for establishing causal relationships due to their random assignment of treatment, minimizing the influence of confounding factors. However, when RCTs are not feasible, alternative analytic methods are used to bridge the gap between observational associations and causal conclusions. Drawing causal conclusions from observational data requires strong additional assumptions, as an observed association between variables can be a result of a true causal mechanism, reverse causation, unmeasured confounding, or sampling bias. Mendelian randomization (MR) offers a method to obtain causal inference based on genetic data and explicit assumptions about the relationship between genetic variables, exposure, and outcome, aiming to eliminate confounding and reduce non-causal associations. A genetic variant can be considered as an instrumental variable (IV) for a given exposure if it satisfies the three basic assumptions: (1) it is associated with the exposure, (2) it is not associated with the outcome due to confounding pathways, and (3) it does not affect the outcome except potentially via the exposure (Greenland, 2000). Genome-wide association study (GWAS) plays a crucial role in MR analysis, as it provides researchers with large-scale genetic data that can be used to identify single nucleotide polymorphisms (SNPs) associated with specific phenotypes or diseases. These SNPs are then used as IVs in MR analysis to assess causal relationships between exposures and outcomes.

Neuroimaging datasets with large numbers of participants allow for population-level exploration of the relationship between disease risk factors, brain structure, function, and disease etiology. The UK Biobank (UKB) stands out as the largest-scale population-based prospective cohort study to date, involving over 500,000 individuals between 40 and 69 years old (Alfaro-Almagro et al., 2018). Its extensive data collection, such as biological samples, physical measurements, and detailed lifestyle questionnaires, makes it the largest resource for genetic and phenotypic data available for academic research. Especially, the UKB has the largest adult brain imaging dataset in over 40,000 participants. The brain imaging protocol has six modalities: T1-weighted structural imaging, resting-state functional MRI, T2-weighted FLAIR structural imaging, diffusion-weighted imaging (DTI), susceptibility-weighted imaging (SWI), and task-based functional MRI (Alfaro-Almagro et al., 2018; Mo et al., 2024). Each modality provides different information, such as brain tissue volume, connectivity between brain regions, microstructural integrity, and functional responses to specific tasks or stimuli. All these modalities end up generating almost 4,000 brain image-derived phenotypes (BIDPs), contributing to a comprehensive understanding of brain structure and function.

While large population imaging studies hold promise in identifying associations between modifiable risk factors and brain phenotypes and diseases risk, the analysis is prone to both meaningful associations and confounding effects, necessitating the need for methods like MR to establish true causal relationships and mitigate hidden confounding. In recent years, there has been a notable increase in the quantity and accessibility of comprehensive neuroimaging GWAS studies. Coupled with the abundant brain imaging data provided by UKB, a significant number of MR studies based on UKB neuroimaging data have emerged, aiming to validate potential causal relationships identified in observational studies. By critically examining the evidence from these MR studies, this work aims to gain a comprehensive understanding of the intricate causal connections involving UKB neuroimaging, and thereby provide important scientific evidence of disease prevention, treatment, and intervention strategies in the field of neuroscience.

## Methods

In accordance with preferred reporting items for systematic reviews and meta-analyses (PRISMA) guidelines, we conducted a systematic review to identify relevant published studies focusing on neuroimaging as either an exposure or outcome. The lead author searched online databases including Pubmed, Scopus, Web of Science and Google Scholar, using keywords such as “neuroimaging,” “MRI,” “UKB,” “UK biobank,” “brain,” “imaging,” “IDP,” “IDPs,” “structure,” “structural,” “function,” “functional,” “MR” and “Mendelian randomization.” No restrictions were applied to the publication date. After the initial literature review, titles and abstracts were screened to exclude studies that did not meet the inclusion criteria. The full text of the remaining manuscripts was then assessed for eligibility. Additionally, reference lists of the included studies were examined to identify any additional relevant publications. Key information was extracted from each manuscript by the lead author, including the racial ancestry of the sample, the number of SNPs used as genetic IVs, F-statistic, odds ratios (ORs) and/or risk ratios (RRs) from MR analysis [with 95% confidence interval (CI)], and *p* value. A majority of manuscripts included a primary MR estimate calculated via inverse-variance weighted (IVW) method, as well as multiple sensitivity analyses to account for potential pleiotropy and heterogeneity, including MR-Egger, Mendelian randomization pleiotropy residual sum and outlier (MR-PRESSO), Cochran’s Q and Leave-one-out tests.

## Results

A total of 34 manuscripts reported MR estimates of the causal relationships involving UKB neuroimaging, primarily focusing on the following aspects: lifestyle and environment, pathological conditions, and biomarkers. Summary of main causal estimates is displayed in Supplementary Table S1.

### Lifestyle and environment

#### Alcohol consumption

For a long time, moderate alcohol consumption (14–21 units/week) was considered harmless or even beneficial, especially in terms of cardiovascular health (Ronksley et al., 2011). However, there is a growing body of research providing evidence that moderate or even light alcohol consumption is associated with health risks (Topiwala et al., 2017). For example, alcohol consumption may induce brain iron accumulation and lead to impaired cognitive function and neurodegenerative diseases (Listabarth et al., 2020). Topiwala et al. investigated the relationship between moderate alcohol consumption and brain iron deposition measured by SWI (Topiwala et al., 2022). They first concluded that moderate alcohol consumption was associated with higher brain iron in multiple basal ganglia regions through observational analyses, and then went on to verify this conclusion through MR analysis. Genetically predicted alcohol consumption was found to have a suggestive positive association with left putamen susceptibility and right hippocampus susceptibility. Genetically predicted alcohol use disorder (AUD) was suggestively associated with higher right putamen susceptibility. These MR findings provide weak evidence for the association between moderate alcohol consumption and higher iron levels in the brain, which represent a potential neural mechanism for alcohol-related cognitive decline.

#### Tea and coffee intake

Tea and coffee are both popular non-alcoholic beverages. Observational studies have suggested the benefits of coffee and tea consumption outweigh the drawbacks, but their effects on the brain are inconclusive (Kim et al., 2015; Poole et al., 2017). Sun et al. conducted an MR study to investigate the relationship between tea intake and several MRI markers closely related to Alzheimer’s disease (AD), including brain volume traits, such as total brain volume, gray matter volume (GMV), white matter volume, left/right hippocampus volume, and cerebral small vessels traits (Sun et al., 2023), such as two commonly-used DTI measures [mean diffusivity (MD) and fractional anisotropy (FA)], white matter hyperintensity (WMH) volume, and brain microbleeds. They found that genetically predicted tea intake was significantly associated with decreased GMV and right hippocampus volume, leading to a decline in language and memory functions. These MR results provide a novel possible mechanism that over-drinking tea might increase the risk of AD by reducing brain volume. Zheng et al. investigated the relationship of coffee consumption and MRI markers of cerebral small vessel disease (CSVD) and brain volume (Zheng and Niu, 2022). They used two phenotypes of coffee consumption: cups of coffee consumed per day, and high or low/no coffee consumption. And they used the same outcome data as in the study by Sun et al. (2023). Cups of coffee consumed per day was found to be inversely associated with GMV, and high coffee consumption was found to be suggestively associated with decreased GMV compared with low/no coffee consumption. These MR findings suggest that genetically predicted excessive coffee consumption is causally associated with reduced GMV of the brain.

#### Tobacco smoking

Numerous observational studies have found that smoking accelerates structural and functional brain decline. Lin et al. performed an MR study to investigate the association of smoking with brain volume (Lin et al., 2023). They used smoking initiation (ever being a regular smoker) as exposure and gray and white matter volume as outcome. There was evidence that genetic liability to smoking initiation was significantly inversely associated with GMV, but not associated with white matter volume. Then the authors further explored the role of smoking on localized GMV. Genetic liability to smoking initiation was found to be significantly correlated with reduced volume of left superior temporal gyrus (anterior division) and right superior temporal gyrus (posterior division). Furthermore, the authors conducted multivariable MR analyses to assess the potential influence of confounding factors on causal effect, and they found that alcohol drinking attenuated the associations between smoking and GMV, suggesting the potential mediating role of alcohol drinking. This study reveals the direct adverse effects of smoking on brain structure and provides new clues for the prevention of related diseases.

In a different MR study, Mo et al. also explored the relationship between tobacco smoking and brain aging (Mo et al., 2023). They built a predictive model for calculating brain age gap (BAG), which represented the difference between predicted brain age and chronological age. They utilized smoking behavioral data from a subset of UKB participants as the exposure and BAG estimates as the outcome instead of directly using neuroimaging data. This study found that smoking status and cigarette per day both had significantly causal effect on BAG. Specifically, the brain age of smokers was 0.21 years older than that of non-smokers, and an extra cigarette per day was associated with an increase in brain age of 0.16 years. This study demonstrate that smoking may accelerate brain aging, which suggests that smoking prevention can be an effective intervention for accelerated brain aging and the age-related cognitive function decline.

#### Social or physical inactivity

Physical activities are associated with a reduced risk of major depressive disorder (MDD) (Schuch et al., 2018), while leisure activities and good social relationships are linked to a reduction in depressive symptoms and prevalence (Schuch et al., 2021; Kaveladze et al., 2022), and these observational findings suggest a bidirectional causal relationship between physical/social activity and MDD. Zhao et al. conducted a bidirectional MR study to clarify this relationship, with BIDPs being considered as potential mediators (Zhao et al., 2023). The study examined 3,144 BIDPs as mediators to investigate the pathway between inactivity and MDD, identifying significant causal effects from inactivity to BIDPs, assessing the causal effect of mediators on MDD risk. The results showed that specific BIDPs, such as GMV in right heschl’s gyrus and volume of third ventricle, partially mediated the positive relationship between leisure/social inactivity and risk of MDD, while surface area in left precentral gyrus, mean orientation dispersion index (ODI) in right sagittal stratum, and mean isotropic of free water volume fraction (ISOVF) in right superior corona radiata partially mediated the positive relationship between physical inactivity and risk of MDD. In addition, the weighted-mean ODI of the left acoustic radiation was found to have a masking effect (the indirect and direct effect showing opposite signs) on the relationship between leisure/social inactivity and MDD, while the volume of the right caudate was found to have a masking effect on the relationship between physical inactivity and MDD. These findings contribute to a deeper understanding of the neural mechanisms by which inactivity affects MDD.

#### Education

Observational neuroimaging studies support the brain reserve hypothesis, showing that higher education levels are associated with increased brain macro-and micro-structural measures implicated in AD, such as GMV, cortical thickness, surface areas of vulnerable regions, and white matter tract integrity (Liu et al., 2012; Kim et al., 2015; Tang et al., 2017). Seyedsalehi et al. employed univariable/multivariable MR to investigate potential causal links between educational attainment (EA), structural brain reserve as indicated by MRI phenotypes, and AD (Seyedsalehi et al., 2023). Genetically predicted EA was found to be inversely associated with AD, while positively associated with cortical metrics such as surface area, volume, intrinsic curvature, and local gyrification index, and inversely associated with cortical intracellular volume fraction and WMH volume. However, there was no evidence of associations between genetically predicted BIDPs and AD, and the inverse association between EA and AD remained largely unchanged even after adjusting for BIDPs, indicating that BIDPs did not act as mediators in the causal pathway from EA to AD. The potential protective effect of higher EA on the risk of AD may be mediated by additional structural brain changes that are not detected by these BIDPs or by alternative biological mechanisms.

#### Vitamin D

Vitamin D has widespread effects on brain health (Maretzke et al., 2020), potentially influencing neurotrophic growth factors, inflammation, and thrombosis. This makes it an attractive candidate for identifying modifiable risk factors for dementia and stroke, as it can be maintained through supplementation, diet, and sunlight exposure (Nitsa et al., 2018). Observational research suggests that low levels of 25-hydroxyvitamin D (25(OH)D) may be associated with cognitive decline and neurocognitive diseases (Annweiler et al., 2014). The potential causal role of 25 (OH) D in brain was assessed by Navale et al. in an observational and MR study (Navale et al., 2022). Through an observational analysis, the association between 25 (OH) D and brain volumes, including total brain, gray matter, white matter, and hippocampal volume, was found to be nonlinear. Both low and high 25 (OH) D concentrations were associated with lower brain volumes for total brain, gray matter, and white matter, while the association with hippocampal volume was only observed at higher concentrations. Then the authors used a genetic risk score (GRS) based on 35 variants to instrument 25 (OH) D concentrations and conducted linear and nonlinear MR analyses, which showed that 25 (OH) D was not associated with any of the neuroimaging outcomes. Larger MR studies are needed to confirm causality for the proposed associations between 25 (OH) D concentrations and brain morphometry.

### Pathological conditions

#### AD

AD is a prevalent neurodegenerative disease characterized by dementia, brain inflammation, and atrophy, affecting millions of people and incurring significant healthcare costs. Recent advancements in MRI have allowed for the identification of physiological brain changes associated with AD, such as volumetric and vascular alterations, connectivity patterns, and white matter damage (Chandra et al., 2019). Genotyping technology and GWAS have also contributed to the discovery of genetic markers associated with AD. Knutson and Pan integrated genetic and MRI-derived biomarkers and aimed to identify significant associations between BIDPs and AD and assess the effectiveness of the tests in detecting genetic associations (Knutson and Pan, 2021). They found that significant possible associations of some BIDPs with AD, including three T1-weighted structural MRI phenotypes related to the calcarine sulcus, WMH volume, thalamic volume, connectivity within various brain regions, and measures of the internal capsule, many of which have well-established links to the characteristic cognitive impairments of AD. In a study assessing the causal relations between BAG and five mental disorders (Leonardsen et al., 2023), Leonardsen et al. found that increased risk of AD was causally associated with increased BAG. The causal effect of genetically predicted risk for AD on BAG was consistently observed across the six MR models. The findings of these two MR studies contribute to a better understanding of the pathogenesis of AD and the mechanisms of brain aging.

#### Stroke

Observational studies have demonstrated certain correlations between stroke and BIDPs. Yu et al. conducted a bidirectional MR study to investigate the causal relationship between 587 BIDPs and different stroke types (Yu et al., 2023). In the forward MR analysis, 14 BIDPs derived from projection or association fibers were found to be causally associated with stroke. These BIDPs included 9 MD values and 1 FA value in different regions. In the reverse MR analysis, genetically determined higher risk of any ischemic stroke was associated with increased ISOVF in the body of the corpus callosum. The reverse MR analysis yielded fewer significant results compared to the forward MR analysis, potentially due to the lower variance explained by the IVs for stroke compared to the IVs for BIDPs. These MR findings provide new perspectives and strategies for early prediction, diagnosis, and treatment of stroke.

#### Psychiatric disorders

Schizophrenia has a substantial genetic contribution, with twin and familial heritability estimates of around 80% and SNPs heritability of approximately 24%. Polygenic risk scores (PRS) constructed from GWAS have been used to investigate the shared genetics between schizophrenia, neurodevelopmental trajectories, and brain morphology (Riglin et al., 2017; Trubetskoy et al., 2022). Stauffer et al. (2021) analyzed genotype and multimodal MRI data from approximately 30,000 participants and measured various MRI metrics and found that higher PRS for schizophrenia was associated with reduced neurite density index (NDI) at a global brain scale, as well as in specific cortical regions, subcortical structures, and white matter tracts. Then they performed exploratory bidirectional MR analyses, examining the causal relationships between schizophrenia and changes in 41 global and regional NDI phenotypes. They found that genetically predicted lower NDI in the thalamus was associated with increased risk for schizophrenia. A study conducted by Guo et al. identified five BIDPs that were causally associated with schizophrenia (Guo et al., 2022). These findings provide insights into the structural brain alterations related to schizophrenia risk. In another study, Mulugeta et al. conducted an MR analysis on six neuroimaging biomarkers, including the volume of the total brain, gray matter, white matter, hippocampus, WMH, and iron deposition on caudate, in order to identify any potential associations with schizophrenia (Mulugeta et al., 2023). However, their findings did not reveal any evidence of a significant association between these biomarkers and schizophrenia.

In addition, three other psychiatric disorders, including bipolar disorder (BD), anorexia nervosa (AN), and depression, have been proven to have causal relationships with neuroimaging measures. An increase in the volume of the left accumbens was associated with a significantly lower risk of BD (Guo et al., 2022). In the reverse direction, increased risk of BD was causally associated with increased BAG (Leonardsen et al., 2023). Three BIDPs located in the superior corona radiata were found to be causally associated with AN (Guo et al., 2022). Depression was found to have a causal effect on lower microstructural integrity in four white matter measures and lower resting-state fluctuation amplitude in the salience network (Shen et al., 2020; Zhang et al., 2024). These MR findings contribute to a better prediction and intervention of psychiatric disorders at the brain imaging level.

#### Migraine

MRI studies have identified widespread abnormalities in the brain during migraine episodes, including both gray matter structural and functional changes (Messina et al., 2022; Zhang et al., 2023). Additionally, DTI studies have shown microstructural alterations in white matter tracts connecting various brain regions (Rahimi et al., 2022). Zhao et al. performed a bidirectional MR study to investigate the causal relationship between microstructural white matter and migraine, and three BIDPs showed significant results (Zhao et al., 2023). The mode of anisotropy (MO) in the left inferior fronto-occipital fasciculus and the OD in the right posterior thalamic radiation had significant causal effects on migraine. Reversely, migraine had a significant causal effect on the OD in the left superior cerebellar peduncle. These findings provide new insights into the role of brain structure in the development and experience of migraine.

#### Cardiovascular diseases

Changes in cardiac function have a significant impact on brain function and may serve as risk factors or biomarkers for brain disorders. Non-organic heart rhythm changes have been identified as potential biomarkers for disorders such as depression and post-traumatic epilepsy, reflecting abnormal brain function (Bahari et al., 2018). Heart diseases such as heart failure and atrial fibrillation (AF) increase the risk of neurological disorders such as dementia and stroke (Doehner et al., 2018). Synchronized heart rhythms and electroencephalography (EEG) signals further confirm the existence of a functional link between the heart and brain (Catrambone et al., 2021). In addition, there are two MR studies that are in favor of the “heart-brain axis.” The first one found a significant association between a higher genetic predisposition for AF and lower GMV (Park et al., 2021). And adjusting for the impact of ischemic stroke weakened the overall influence of AF on lower GMV, indicating that ischemic stroke may act as a mediator in the causal pathway. The other found that two genetically predicted traits of heart rate variability, the root mean square of the successive differences of inter beat intervals and the peak-valley respiratory sinus arrhythmia or high frequency power were suggestively positively associated with WMH volume (Tian et al., 2021).

There is observational evidence suggesting that decreased aortic elasticity, measured as a decrease in distensibility, predicts diseases such as CSVD and AD (van Sloten et al., 2015). In addition, aortic stiffness is more useful than blood pressure in predicting WMH volume (King et al., 2013). Francis et al. conducted a study to explore the genetic associations underlying these relationships (Francis et al., 2022). The authors derived six aortic traits (ascending/descending aortic distensibility, maximum/minimum ascending aortic area, and maximum/minimum descending aortic area) from cardiac MRI images, which subsequently were used in MR analyses to investigate the potential causal relationships between different aortic traits and WMH volume. The multivariable MR analysis suggested a genetically causal correlation between decreased aortic distensibility or increased ascending aortic area and increased burden of WMH.

These MR results provide causal links between cardiovascular disease and brain imaging traits, highlighting potential risk factors for related brain health issues.

#### Glycemia and diabetes

Observational studies suggest an association between hyperglycemia, diabetes, insulin resistance, and deteriorating brain health, including worse cognitive function, risk of cognitive decline, dementia, and CSVD (Brundel et al., 2014; Xue et al., 2019). Liu et al. performed MR analyses to test the causal relationship of type 2 diabetes (T2D), fasting glucose, and fasting insulin with two clinical outcomes associated with CSVD (MRI-confirmed lacunar stroke and intracerebral hemorrhage) and three radiological markers of CSVD (WMH, FA and MD) (Liu et al., 2018). T2D was found to be associated with a higher risk of lacunar stroke and lower mean FA, indicating potential negative effects on brain health. No significant associations were observed between T2D and WMH volume, mean MD, intracerebral hemorrhage. Garfield et al. conducted another MR study to explore the causal effects of T2D or glycated hemoglobin (HbA_1c_) on cognitive function, structural brain measures such as hippocampal volume and WMH volume, and AD (Garfield et al., 2021). The results showed that T2D and HbA_1c_ were not associated with either of these two measures, which suggested that these associations were not likely to be causal. The discrepancy of the results may be due to different exposure and outcome, further investigations with larger sample size are required to identify the causal relationship.

#### Poor oral health

Observational studies have linked poor oral health, specifically periodontitis and tooth loss, to higher risks of cognitive decline, dementia, and stroke (Fagundes et al., 2020; Asher et al., 2022). Rivier et al. conducted a two-stage study to investigate the relationship between oral and brain health (Rivier et al., 2023). The first stage, consisting of a series of epidemiological analyses, showed that poor oral health was significantly associated with a 9% increase in WMH volume, a 10% change in aggregate FA or MD score, and a 10% change in aggregate MD score. These findings were confirmed in the second stage, where several MR approaches were employed. At the global level, genetically determined poor oral health was associated with a 30% increase in WMH volume, a 43% change in aggregate FA score, and a 10% change in aggregate MD score. At the regional level, genetically determined poor oral health was associated with FA or MD changes in most of the regions, especially the retrolenticular part of the internal capsule. These results suggest that genetically determined poor oral health is associated with extensive microstructural damage.

#### Bone mineral density and osteoporosis

Osteoporosis is becoming more common as the global population ages, and it is characterized by a reduction in bone mineral density (BMD), increasing the risk of fractures. The central nervous system plays a role in regulating bone mass and there is a neural connection between the brain and bone (Maryanovich et al., 2018). Observational evidence supports that neurodegenerative diseases are associated with a higher prevalence of osteoporosis (Raglione et al., 2011; Simonsen et al., 2016; Caplliure-Llopis et al., 2020; Kumar et al., 2021). Guo et al. conduct a study to investigate the genetic correlations and causal associations between 1,325 BIDPs and BMD at 5 different locations (total body, lumbar spine, femoral neck, forearm, and heel) (Guo et al., 2023). As a first step, linkage disequilibrium score regression (LDSC) was used to identify 1.93% of BIDPs showing significant genetic association with BMD. And the following MR analyses revealed 1.31% of BIDPs exhibited a significant causal association with BMD. Interestingly, their results showed that more left BIDPs were causally associated with BMD, especially within and around the left frontal region. The confirmatory MR between the BIDPs and osteoporosis showed that most of the BIDPs’ potentially causal associations with BMD and osteoporosis demonstrated opposite directions. Overall, these findings indicate that alterations in brain structure could impact bone metabolism through specific pathways, emphasizing the need for further research on the genetic mechanism of the brain-bone axis to enhance the prediction and prevention of osteoporosis.

#### Ankle sprain

Resulting from acute ankle sprains, chronic ankle instability can have persistent effects on individuals (Gribble et al., 2013). In addition to biomechanical issues, neural inhibition of the surrounding muscles may affect motor control and function (Hertel and Corbett, 2019). Some studies suggest that ankle sprains can lead to impairments in the corticospinal tract (CST) pathway, which in turn can result in muscular dysfunction (Terada et al., 2016; Kosik et al., 2017). CST damage could be manifested as changes in DTI parameters, including a decrease in FA and an increase in ODI. To establish the causal relationship between ankle sprains and damage to the CST, Xue et al. conducted an MR study using FA and ODI values of the left and right CST as the outcome (Xue et al., 2023), and found ankle sprains were associated with a decrease in FA of the right CST and an increase in ODI of both sides of CST. The association between ankle sprains and the increase in ODI in the left CST was significant, and the other two were suggestive. These findings support that ankle sprains could trigger abnormal organization of CST neurites.

### Biomarkers

#### Blood pressure

Elevated blood pressure (BP) is linked to cerebrovascular diseases and dementia (Collaborators, 2018), and observational studies and clinical trials suggest a causal relationship between hypertension and cognitive function (SPRINT MIND Investigators for the SPRINT Research Group et al., 2019). However, the exact mechanisms and specific areas involved in this relationship have been challenging to determine. Several studies used MR methods to identify brain structures potentially associated with BP values. Taylor-Bateman et al. chose 3 neuroimaging features of CVSD (WMH, FA and MD measures for 48 regions) as outcomes and found robust evidence supporting a causal association between BP traits and all three features (Taylor-Bateman et al., 2021). Moreover, diastolic blood pressure (DBP) was found to be the main contributing factor, instead of systolic blood pressure (SBP). Ye et al. concentrated on FA measures for 39 regions and identified significantly negative causal effects of BP on 21 and 18 WM tracts for SBP and DBP, respectively (Ye et al., 2023). Siedlinski et al. conducted a comprehensive assessment, integrating data on SBP, DBP and pulse pressure (PP) with 3,935 BIDPs (Siedlinski et al., 2023). According to the MR analysis, 242, 168, and 68 BIDPs were significantly associated with SBP, DBP and PP. And most of the identified associations aligned with findings from previous observational studies. Kolbeinsson et al. trained a deep convolutional neural network model to predict brain age based on T1-weighted images, and then calculated the brain age difference (similar to BAG) by comparing it with the actual age (Kolbeinsson et al., 2020). Subsequently, MR analyses were conducted to investigate the relationship between a range of factors and brain age difference. The results indicated the association of a higher genetically determined DBP with higher brain age difference. Similarly, Feng et al. evaluated the relationship between BP and BAG using MR and obtained consistent results: there was an overall significant positive causal relationship between DBP and BAG (Feng et al., 2023). The results of these MR studies contribute to a better understanding of the mechanisms by which hypertension affects brain health.

#### LDL-C

Lipid-lowering medications are widely used to manage vascular diseases by controlling cholesterol levels in the blood. However, some lipid-lowering drugs, such as PCSK9 inhibitors, have various effects, including reducing the risk of cancer (Carter et al., 2020) and increasing the risk of type 2 diabetes (Rao et al., 2018). Pham and colleagues conducted a study aiming to explore possible associations between low density lipoprotein cholesterol (LDL-C) lowering and various diseases or biomarkers (Pham et al., 2023). The authors selected SNPs within 100 KB on either side of four gene regions associated with LDL-C (PCSK9, HMGCR, NP1L1, and LDLR) and 52 serum, urine, imaging, and clinical biomarkers including brain volume data as the outcomes in the MR analyses. The results showed that PCSK9 was suggestively associated with higher WMH volumes, while HMGCR was significantly associated with slightly higher hippocampal volume. Further research is needed to confirm and explain these associations.

#### IL-6/IL-6 receptor

Interleukin-6 (IL-6) and its receptor IL-6α, which can cross the blood–brain barrier and increase its permeability (Upthegrove and Khandaker, 2020), have been associated with changes in brain structure and the development of neuropsychiatric disorders (Roohi et al., 2021). Williams et al. investigated the potential causal relationship between inflammatory cytokines including IL-6 (Williams et al., 2022). In the MR analyses, five inflammatory cytokines (IL-1, IL-2, IL-6, C-reactive protein, and brain-derived neurotropic factor) were used as exposures, and regional cortical thickness and brain volume measures were used as outcomes. Genetically predicted IL-6 levels were found to be linked to GMV in the middle temporal cortex, inferior temporal cortex, fusiform cortex, and frontal cortex, as well as cortical thickness in the superior frontal region. This study provides ample evidence for a causal association between IL-6 and structural changes in the cortex.

#### Telomere length

Telomeres, DNA-protein complexes which maintain genome stability, shorten with each cell division but can be replenished by telomerase enzyme activity. Critically short telomere length (TL) can trigger cellular senescence and a pro-inflammatory state, potentially impacting various organs including the brain (Kuo et al., 2019). Liu et al. conducted an observational and MR study to assess associations of leukocyte TL with AD and AD-related dementias (ADRD), as well as early indicators of AD/ADRD such as cognitive performance and 62 BIDPs associated with dementia (Liu et al., 2023). Longer TL was found to be significantly associated with higher FA and lower MD in several tracts. Salih et al. also assessed the causal relationships between TL and BIDPs, but in a more comprehensive and systematical way where 3,935 BIDPs were tested (Salih et al., 2022). A total of 193 BIDPs were found to be significantly influenced by TL, the majority of which were related to white matter. Interestingly, the direction of TL-BIDP associations did not align with the direction of changes associated with aging. For example, previous studies showed that FA declined with aging, but FA was found to be decreased as TL increased in this study. Overall, this MR analysis suggests that the relationship between TL and BIDPs is more complex than simply reflecting increased cellular aging.

#### Metabolomics

Metabolomics allows for detailed quantification of small metabolic markers in biological samples to discover new disease biomarkers and understand the metabolic pathways underlying diseases. Previous metabolomics studies have identified metabolites associated with CSVD, cerebral microbleeds, and dementia. In a large-scale metabolomics study conducted by Harshfield and Markus (2023), they analyzed the relationships of 325 metabolites with MRI markers of CSVD (WMH, FA, and MD), stroke, and dementia, and assessed the causality of these relationships using MR approach. They found 289 metabolic traits significantly associated with MRI markers of CSVD and future risk of stroke and dementia. However, only a few of these associations, primarily in relation to late-onset AD, were validated as significant causal relationships by MR analysis. In addition, some metabolic traits were found to have suggestive causal relationships with MRI markers of CSVD. It is worth noting that these metabolites showed stronger correlations with DTI parameters compared to the WMH volume. Their study provides evidence supporting the association of a wide range of metabolites with stroke, dementia, and MRI markers of SVD.

## Discussion

The 34 MR studies included in this paper have investigated the genetically causal relationships between lifestyles/biomarkers and brain imaging, and between diseases and brain imaging. Understanding the causal relationships between lifestyles and brain imaging can raise people’s awareness of the impact of lifestyle choices on brain health, and encourage them to take proactive actions and pursue a healthier lifestyle to promote brain health. Understanding the causal relationships between diseases and brain imaging not only contributes to a better understanding of the pathophysiology of neurological disorders but also reveals the comprehensive impact of diseases on the whole body, fostering interdisciplinary collaboration and providing more effective approaches for comprehensive disease management. And understanding the causal relationship between biomarkers and brain imaging allows for the identification of new biomarker indicators for early disease prediction and diagnosis, facilitating personalized treatments.

MR studies have distinct advantages over observational studies by reducing biases through the use of genetic variants as IVs. MR enables stronger causal inference by leveraging genetic variants assigned at conception, overcoming limitations in establishing causality. Additionally, MR minimizes reverse causation, mitigates measurement errors, and allows for replication and generalizability of findings, providing robust evidence for understanding the relationships between exposures and outcomes. MR can achieve genetic evidence of causal results with clear direction, thereby providing a reliable basis for delineating the etiology and specific guidance for disease prevention. Compared to RCTs, MR has better operability as it can utilize existing public databases, such as UKB.

However, the 34 MR studies involving UKB brain imaging have several common limitations. Firstly, although UKB is currently one of the largest long-term health research projects, the quantity of its brain imaging data remains relatively insufficient. This insufficiency may lead to an imbalance in sample sizes between the exposure and the outcome in MR studies. Additionally, UKB predominantly consists of individuals of European descent. As genetic variants and their impact on health outcomes can vary across populations due to factors like evolutionary history and environmental exposures, caution must be exercised when extrapolating study results to populations with different genetic backgrounds. Future MR studies should include diverse cohorts representing different races and ancestries, and utilize more comprehensive brain imaging data, to improve the applicability and generalizability of research findings and achieve a comprehensive understanding of the relationship between genetic variants and health outcomes across diverse populations. Secondly, data overlap occurred in most of the studies. In two-sample MR studies, IVs are derived from one dataset, representing genetic variants associated with the exposure of interest, while the outcome is evaluated using summary statistics from another dataset. In an ideal scenario, these datasets would be independent and non-overlapping, ensuring the robustness of the findings. However, in practice, it is challenging to completely avoid data overlap when using publicly available aggregated data. Data overlap can introduce potential bias and affect the validity of the results, potentially leading to inflated effect estimates or violations of MR assumptions. Based on the Burgess simulation (Burgess et al., 2016), the proportion of data overlap in any of the included MR studies was not significant enough to cause a type 1 error. Thirdly, while these MR studies have established certain causal relationships, there remains a lack of comprehension regarding the underlying biological mechanisms driving these associations. Although MR provides evidence for causality, its reliability is not as strong as RCT, and it does not inherently reveal the specific pathways or biological processes at play. Therefore, additional research, such as animal experiments, is necessary to further validate causal relationships and explore the biological mechanisms through which these genetic variants and exposures influence the outcomes of interest. This may involve in-depth analysis of molecular and cellular components, functional genomics, and pathway examination, all aimed at uncovering the molecular cascades and biological pathways responsible for mediating the observed causal effects. A thorough understanding of these mechanistic connections can yield crucial insights into disease etiology and potential therapeutic targets. By integrating MR with other genomic and functional approaches, comprehensive investigations can be undertaken to bridge the gap between causal relationships and biological mechanisms. Ultimately, these efforts will enhance our comprehension of complex diseases and facilitate the development of targeted interventions.

## Conclusion

The 34 MR studies involving UKB brain imaging primarily focus on the causal relationship between lifestyle and environment, pathological conditions, and biomarkers, and BIDPs. In terms of lifestyles and environment, alcohol consumption is associated with iron deposition in certain areas of the brain. High tea/coffee intake and smoking are associated with a reduced volume in certain areas of the brain. Education attainment is positively correlated with some brain morphological characteristics and negatively correlated with others. And some BIDPs can serve as mediators between leisure/physical inactivity and MDD. However, vitamin D has no causal relationship with brain morphological characteristics. In terms of diseases, there are causal relationships between BIDPs and neurological diseases like AD, stroke, and migraines, as well as several psychiatric disorders. Furthermore, BIDPs are also associated with diseases of other systems such as cardiovascular diseases, diabetes, poor oral health, osteoporosis, and ankle sprains. In terms of biomarkers, BIDPs are associated with BP, LDL-C, IL-6, TL, and more.

## Author contributions

MW: Writing – original draft. ZW: Writing – original draft. YW: Writing – original draft, Writing – review & editing. QZ: Writing – review & editing. JW: Writing – review & editing.
